# The sotos syndrome gene Nsd1 safeguards developmental gene enhancers poised for transcription by maintaining the precise deposition of histone methylation

**DOI:** 10.1016/j.jbc.2025.108423

**Published:** 2025-03-19

**Authors:** Jie Li, Zhucui Li, Jiekai Yin, Yinsheng Wang, Deyou Zheng, Ling Cai, Gang Greg Wang

**Affiliations:** 1 Division of Hematology and Oncology, Department of Medicine, Weill Cornell Medicine, Cornell University, New York, New York, USA; 2 Department of Biochemistry, Weill Cornell Medicine, Cornell University, New York, New York, USA; 3 Environmental Toxicology Graduate Program and Department of Chemistry, University of California Riverside, Riverside, California, USA; 4 Department of Genetics, Albert Einstein College of Medicine, Bronx, New York, USA; 5 Department of Neurology and Department of Neuroscience, Albert Einstein College of Medicine, Bronx, New York, USA; 6 Department of Pathology, Duke University School of Medicine, Durham, North Carolina, USA; 7 Department of Pharmacology and Cancer Biology, Duke University School of Medicine, Durham, North Carolina, USA

**Keywords:** development, differentiation, gene enhancer, H3K27ac, H3K27me3, H3K36me2, Nsd1, sotos syndrome

## Abstract

Germline haploinsufficiency of NSD1 is implicated as the etiology of Sotos syndrome; however, the underlying mechanism remains far from being clear. Here, we use mouse embryonic stem cell (mESC) differentiation as a model system to address this question. We found Nsd1 to be indispensable for the faithful differentiation of mESCs into three primary germ layers, particularly, meso-endodermal cell lineages related to the development of the heart and the skeletal system. Time-course transcriptomic profiling following the mESC differentiation revealed that Nsd1 not only facilitates the basal expression but also permits the differentiation-accompanied rapid induction of a suite of meso-endoderm lineage-specifying transcription factor genes such as *T* and *Gata4*. Mechanistically, Nsd1 directly occupies putative distal enhancers of the lineage transcription factor genes under the pluripotent cell state, where it deposits H3K36me2 to antagonize the excessive H3K27me3 and maintains the basal H3K27ac level, thereby safeguarding these gene enhancers at a primed state that responds readily to differentiation cues. In agreement, gene rescue assays using the Nsd1 KO mESCs showed that the H3K36me2 catalysis by Nsd1 requires several functional modules within Nsd1 (namely, PHD1-4, PWWP2, and SET) to a similar degree. Disruption of either one of these Nsd1 modules severely abrogated H3K36me2 in mESCs and significantly impaired appropriate induction of developmental genes upon mESC differentiation. Altogether, our study provides novel molecular insight into how the NSD1 perturbation derails normal development and causes the disease.

Sotos syndrome, an autosomal dominant genetic disorder, is characterized by a range of variable clinical features such as distinctive facial appearance, learning disability, childhood overgrowth, congenital cardiac and renal defects, advanced bone age, and seizure and scoliosis (1, 2, 3). Germline heterozygous pathogenic variations of the nuclear receptor-binding SET domain-containing protein 1 (NSD1, also known as KMT3B), notably the gene truncation and missense mutations, are detected in more than 90% of patients with Sotos syndrome and thus nominated as the main causal lesion of this disease (4, 5, 6, 7). The prevalence of NSD1 mutations in Sotos syndrome patients also suggests a critical involvement of NSD1 in the regulation of normal development. In mice, Nsd1 was documented to be essential for appropriate gastrulation during early post-implantation development (8). However, the molecular mechanisms underlying the NSD1-directed (epi)genomic regulation during development remain largely unclear to date.

NSD1, along with NSD2 (also known as MMSET) and NSD3 (also known as WHSC1L1), constitutes the NSD family of histone lysine methyltransferases that use a conserved catalytic domain termed Su(var)3 to 9, Enhancer-of-zeste and Trithorax (SET), to selectively mediate mono- and di-methylation of histone H3 lysine 36 (H3K36me1 and H3K36me2) (9, 10). Unlike H3K36me3 that is predominantly enriched at body regions of the actively transcribed gene, H3K36me2 spreads widely across the genome, including the intergenic and genic regions (11). A growing body of evidence suggests that H3K36me2 elicits its (epi)genome-regulatory effects at least partly through its crosstalk with cellular machineries mediating tri-methylation of histone H3 lysine 27 (H3K27me3) and DNA methylation (11, 12). In various normal and diseased settings such as mouse embryonic stem cells (mESCs) and acute myeloid leukemia, the NSD1-deposited H3K36me2 was shown to antagonize and restrict the spreading of polycomb repressive complex 2 (PRC2)-mediated H3K27me3 (13, 14, 15, 16). On the other hand, H3K36me2 can be recognized and bound directly by the Pro-Trp-Trp-Pro (PWWP) domain of DNA methyltransferase 3A (DNMT3A), thereby directing the *de novo* DNA methylation across the broad intergenic regions (15, 17).

In addition to the intrinsic H3K36-methylating activity harbored in the SET domain, NSD1 also contains several protein modules, such as two nuclear receptor interaction domains (NIDs; which contain the so-called LXXLL motif, where L is leucine and X can be any amino acid) in the N-terminal region (18), two PWWP domains (PWWP1 and PWWP2, with the former only present in the longer isoform of NSD1), as well as five classical PHD finger domains (PHD1 to PHD5) and one atypical C5HCH-type PHD finger that are clustered around the SET domain. Previous studies have linked these domains of NSD1 to its various interactions with histone, nonhistone protein partner, DNA, or RNA (19, 20, 21). However, relevance of these protein modules to NSD1’s function remains to be carefully investigated under different biological contexts. In fact, the Sotos syndrome-associated NSD1 missense mutations are enriched at a C-terminal region that encodes the NSD1 PHD1-4, PWWP2, SET, and PHD5-C5HCH (5, 22), highlighting a functional significance of these domains.

Here, we employ mESCs as a model system to systematically dissect the role for Nsd1 in regulating cell differentiation and lineage specification, which provides new insights into how the Nsd1 perturbation results in wide-spread abnormalities in normal development, implicative of the molecular mechanism underlying pathogenesis of the Sotos syndrome.

## Results

### Nsd1 preferentially binds the poised enhancers of developmental genes in mESCs

**Figure 1:**
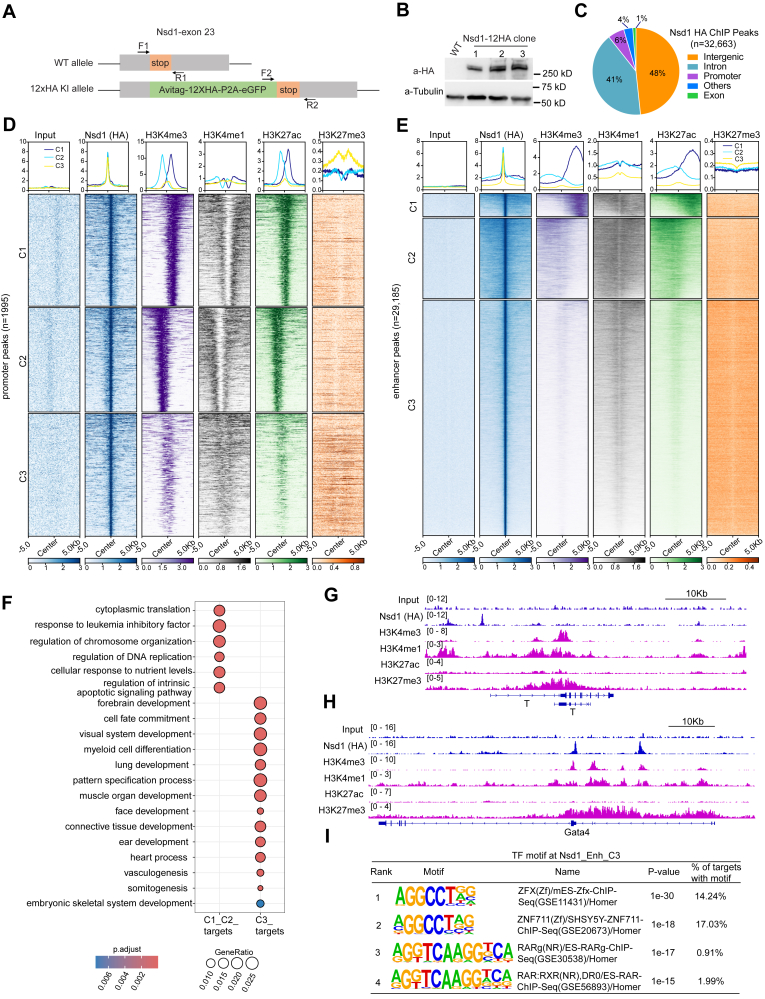
**Nsd1 mainly binds the poised enhancers of developmental genes in mESCs.***A*, a schematic illustrating knock-in (KI) of an AviTag-12×HA-P2A-eGFP cassette in-frame to the C-terminus of Nsd1 gene. *Arrows* indicate the location of RT-qPCR primers used in Fig. S1, *G* and *H*. *B*, immunoblotting for endogenous, 12×HA-tagged Nsd1 in the E14 mESCs. Untagged parental cells were used as a negative control. *C*, pie chart showing genomic feature distribution of the called Nsd1-12×HA ChIP-seq peaks. Other features include 5′-UTR, 3′-UTR, miRNA, ncRNA, transcription termination site (TTS), and pseudo-gene. *D* and *E*, averaged signal profiles and K-means-clustered heatmaps displaying the indicated ChIP-seq signals over 10 kb regions centered around the called Nsd1-bound promoter peaks (*D*, defined as -1kb to 100 bp around transcription start site [TSS]), or the Nsd1-bound putative enhancer peaks (*E*, including intergenic and intronic peaks), in mESCs. Panel *D*: n = 659, 614, and 722 peaks for clusters C1, C2, and C3, respectively. Panel *E*: n = 1,990, 6934, and 20,261 peaks for clusters C1, C2, and C3, respectively. *F*, dot plot showing the indicated gene ontology (GO) terms enriched among target genes associated with the Nsd1-bound C1+C2 enhancers (n = 3119 genes) *versus* C3 enhancers (n = 7984 genes). Dots are differentially colored and sized based on their corresponding BH-adjusted *p* values and gene ratio values, respectively. *p* values were calculated by hypergeometric test and BH-adjusted for multiple comparisons. *G* and *H*, Integrative Genomics Viewer (IGV) tracks showing the reads per genomic content (RPGC)-normalized ChIP-seq signals of the indicated factor at the Nsd1-bound C3 enhancer target genes, *T* (G) and *Gata4* (H). *I*, summary of the top four most enriched TF motifs at the Nsd1-bound C3 enhancer peaks. Motif enrichment was statistically determined by ZOOPS scoring (zero or one occurrence per sequence) coupled with the hypergeometric enrichment calculations. ChIP-seq, chromatin immunoprecipitation sequencing; mESC, mouse embryonic stem cell; NSD1, nuclear receptor-binding SET domain-containing protein 1; RT-qPCR, reverse transcription followed by quantitative PCR; TF, transcription factor.

In mESCs, Nsd1 is expressed at a significantly higher level than Nsd2 and Nsd3 (Fig. S1*A*), indicating that mESCs represent a suitable model for studying the Nsd1 function during development. We first aimed to map the genome-wide distribution of endogenous Nsd1 in mESCs; however, none of the tested commercial antibodies of Nsd1 worked for chromatin immunoprecipitation sequencing (ChIP-seq) in our hands. To address this issue, we used a CRISPR-Cas9-based editing approach to knock-in (KI) either a 3×Flag-P2A-neo or AviTag-12×HA-P2A-eGFP cassette in-frame to the C terminus of endogenous Nsd1 gene (Figs. 1*A* and S1*B*). Single cell-derived E14 mESC clones carrying homozygous KI of the cassette were first identified based on genotyping (Fig. S1, *C*–*F*) and then confirmed by Sanger sequencing of PCR-generated amplicon (data not shown), reverse transcription followed by quantitative PCR (RT-qPCR) (Fig. S1, *G* and *H*), as well as Western blot for the tagged full-length Nsd1 with tag-specific antibody (Fig. 1*B*). Independently derived clonal lines of mESCs were used for subsequent experiments.

Next, we performed ChIP-seq for endogenous Nsd1 using either Flag or hemagglutinin (HA) antibody in two independent mESC lines carrying KI of the respective tag at the Nsd1 gene. Peak calling (with a cutoff set to q value less than 0.05) showed that ChIP-seq signals of Nsd1-12×HA were more robust and yielded more Nsd1 peaks (n = 32,682 peaks; Table S1) than those of Nsd1-3×Flag (n = 9097 peaks), which is likely due to a difference in the number of tag repeats (12×HA *versus* 3×Flag). For this reason, we focused on the Nsd1-12×HA ChIP-seq data for downstream analysis. Genomic feature annotation showed that the Nsd1 peaks are mostly enriched at the intergenic (approximately 48% of all peaks) and intronic (41%) regions, with only a small fraction being at gene promoters (6% of peaks), suggesting a putative regulatory role of Nsd1 predominantly at gene enhancers (Fig. 1*C*). To better understand epigenomic features of the called Nsd1 peaks, we split them to promoter-associated peaks (n = 1995, located -1kb to +100 bp from transcriptional start site [TSS]) or putative enhancer-associated peaks (n = 29,185, including intergenic and intronic regions), followed by assessment of their respective chromatin states by integrated analysis of a suite of histone modifications that define promoter and enhancer states, namely, H3K4me3, H3K4me1, H3K27ac, and H3K27me3 (23, 24, 25). In general, H3K4me3 and H3K4me1 exhibit high abundance, respectively, at gene promoters and enhancers; additionally, H3K27ac and H3K27me3 further define those active and poised/bivalent promoters/enhancers, respectively (23, 24, 25). K-means clustering separated the Nsd1-bound promoter peaks (Fig. 1*D*) and Nsd1-bound enhancer peaks (Fig. 1*E*) into three distinctive clusters (referred to as C1, C2, and C3), with C1 and C2 clusters corresponding to an active state (that is, high for H3K4me3 or H3K4me1, high for H3K27ac, and low for H3K27me3), and C3 corresponding to a poised bivalent state (that is, intermediate-to-low for H3K4me3 or H3K4me1, low for H3K27ac, and high for H3K27me3) (Fig. 1, *D* and *E*). Unlike the promoter regions where Nsd1 displays comparable bindings among the above three clusters, the active enhancer regions (C1+C2) contain the higher levels of Nsd1 binding than the poised enhancers (C3), highlighting Nsd1 as a positive regulator of the gene enhancer activity. Given that the Nsd1-bound enhancer peaks are disproportionately more enriched at the C3 bivalent enhancers (Fig. 1*E*, n = 20,261 which accounts for about 62% of all peaks), we speculate that a major function of Nsd1 in mESCs is to regulate activity of the poised, bivalent gene enhancers.

Next, we assigned the Nsd1-bound enhancer peaks to target genes based on closest TSS proximity, followed by gene ontology (GO) enrichment analysis. Here, the Nsd1-bound active enhancers (C1 and C2 peaks, n = 3119 genes) are mainly associated with genes involved in stemness maintenance such as Pou5f1 (also known as Oct4), Nanog, and Nr5a2, as well as cell proliferation and survival such as ribosomal protein genes and genes related to leukemia inhibitory factor (LIF) response and DNA replication (Figs. 1*F*, S1, *I*–*K*; Table S1). In contrast, genes associated with the Nsd1-bound bivalent enhancers (C3 peaks, n = 7984 genes) are mostly enriched with genes controlling embryogenesis such as those related to the development of heart, lung, muscle, and blood cells (*e.g.*, T, Gata4, Nfatc2, and Hand1) (Figs. 1, *F*–*H*, S1, *L* and *M*). Furthermore, the motif search analysis of Nsd1-bound enhancer peaks revealed enrichment for the consensus binding motifs of a suite of transcription factors (TFs) including ZFX, ZNF711, RAR, and RXR, irrespective of enhancer clusters or types (Figs. 1*I* and S1*N*). These data suggested that Nsd1 is likely recruited to its target genomic loci by these TFs.

Together, the above results support Nsd1 to be a putative enhancer activator, which preferentially binds those poised bivalent enhancers that are involved in controlling key developmental genes in mESCs. Nsd1 may function to prime the lineage-specifying genes in a poised state in the pluripotent stem cells, allowing their rapid induction upon receiving differentiation cues.

### Nsd1 is essential for *in vitro* differentiation of mESCs

**Figure 2:**
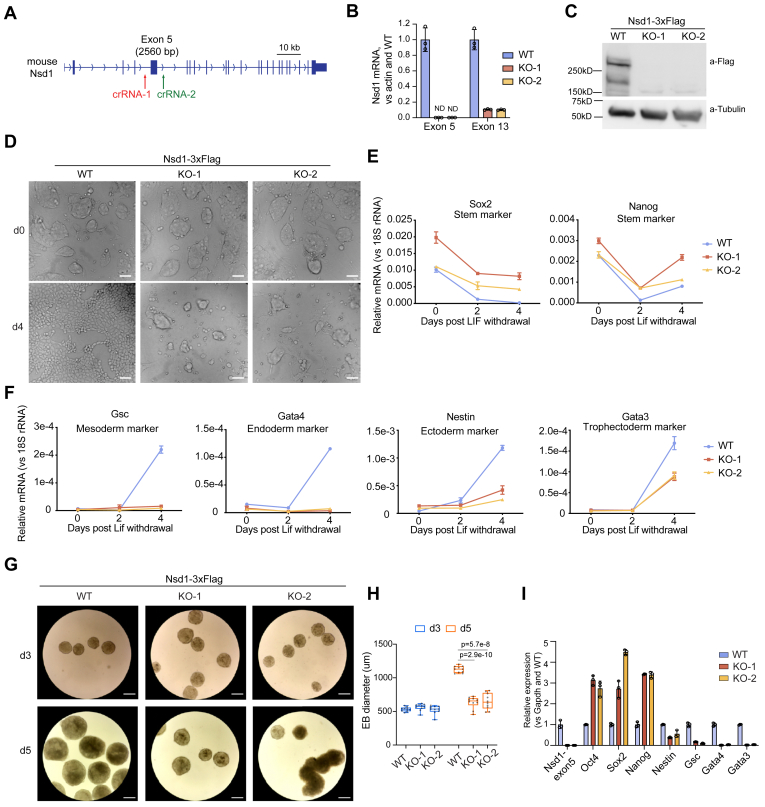
**Nsd1 is indispensable for *in vitro* differentiation of mESCs.***A*, a schematic showing the mouse Nsd1 gene structure and its KO strategy. Two CRISPR RNAs (crRNAs) flanking the mouse Nsd1 exon5 were simultaneously transduced for the targeted genomic deletion. *B*, RT-qPCR using primers specific to exon 5 (*left*) or exon 13 (*right*) of Nsd1 in mESCs with WT Nsd1 or homozygous Nsd1 KO (KO-1 and KO-2 as two independent lines). PCR signals from three independent experiments were normalized to those of actin, and then to WT, and then presented as mean ± SD. *C*, immunoblotting for endogenous Nsd1 in mESCs, which carry WT Nsd1 with in-frame KI of a 3×Flag tag (lane 1) or the Nsd1 KO (lanes 2–3). *D*, representative images showing the morphology of WT and Nsd1-KO mESC cultures, either before (*top*) or 4 days (*bottom*) after withdrawal of LIF and MEFs in a monolayer differentiation protocol. The scale bar represents 500 μm. *E* and *F*, RT-qPCR of the indicated pluripotency (*E*) or lineage marker genes (*F*) during the monolayer differentiation of mESCs. PCR signals from three independent experiments were normalized to those of 18S rRNA and presented as mean ± SD. *G* and *H*, representative images (*G*; the scale bar represents 500 μm) and statistics of diameter distribution (*H*; n = 10, measured by ImageJ) using the WT and Nsd1-KO embryoid bodies (EBs) at day 3 or day 5 of the mESC differentiation. The *p* values in (*H*) were calculated by two-sided Student’s *t* test. *I*, RT-qPCR analysis of the indicated gene expression in the WT *versus* Nsd1-KO EBs, collected at day 5 post differentiation. PCR signals from three independent experiments were normalized to those of Gapdh and then WT and presented as mean ± SD. EB, embryoid body; KI, knock-in; MEF, mouse embryonic fibroblast; LIF, leukemia inhibitory factor; mESC, mouse embryonic stem cell; NSD1, nuclear receptor-binding SET domain-containing protein 1; RT-qPCR, reverse transcription followed by quantitative PCR.

Given a marked enrichment of Nsd1 at the bivalent gene enhancers, we next queried whether Nsd1 plays a critical role in the regulation of mESC differentiation. Toward this end, we conducted CRISPR-Cas9-based KO of Nsd1 in mESCs carrying the Nsd1-3×Flag KI alleles (Fig. 2*A*). Specifically, two guide RNAs flanking the exon 5 of Nsd1 were simultaneously introduced to induce the Nsd1 KO. After genotyping-based screen of single cell clones, we identified independent lines with the homozygous KO of Nsd1 (Fig. S2*A*), which were further validated by RT-qPCR and Western blot (Fig. 2, *B* and *C*). In agreement with previous reports (14, 17), Nsd1 KO led to a global decrease of both H3K36me2 and DNA methylation, as well as a concomitant global increase of H3K27me3 (Fig. S2, *B* and *C*). These observations not only show that Nsd1’s functionalities cannot be compensated by those of Nsd2 or Nsd3 in mESCs but also support a notion that Nsd1 has a predominant role in modulating the chromatin modification landscape, notably, a balanced distribution of H3K36me2, DNA methylation, and H3K27me3.

Next, we removed both LIF, a mESC supporting cytokine, and the feeder mouse embryonic fibroblasts (MEFs) to induce the mESC differentiation using a protocol involving either the two-dimensional (2D) monolayer cell cultures or the hanging drop-based formation of 3D spherical embryoid bodies (EBs) (26). At day four after the removal of LIF and MEFs from the monolayer mESC cultures, the Nsd1-WT cells were almost fully differentiated and manifested a typical flattened appearance, whereas those with Nsd1-KO still formed the ESC-like colonies, suggestive of a delayed differentiation (Fig. 2*D*). In support, these ESC-like Nsd1-KO colonies remained to be alkaline phosphatase (AP)-positive even 6 days post induction of differentiation (Fig. S2*D*). RT-qPCR showed that both the differentiation-accompanied silencing of the classic pluripotent genes (such as Sox2 and Nanog) and the induction of the cell lineage differentiation marker genes, especially those known to specify the mesoderm and endoderm layers (such as Gsc, Gata4, Nestin, and Gata3), were significantly impaired by Nsd1 loss (Fig. 2, *E* and *F*). Similar defective differentiation phenotypes were evident in the EB formation assays, in which the Nsd1-KO EBs manifested the markedly smaller sizes when compared with their WT counterparts 5 days after the withdrawal of LIF and MEFs (Fig. 2, *G* and *H*). Likewise, the Nsd1-KO EBs also expressed the pluripotent genes at higher levels and expressed the lineage marker genes at lower levels, when compared with the Nsd1-WT EBs at day 5 following the differentiation induction (Fig. 2*I*).

Taken together, we show Nsd1 to be essential for appropriate differentiation of mESCs *in vitro*.

### Nsd1 KO impairs appropriate induction of primary germ layer-specifying genes during differentiation

**Figure 3:**
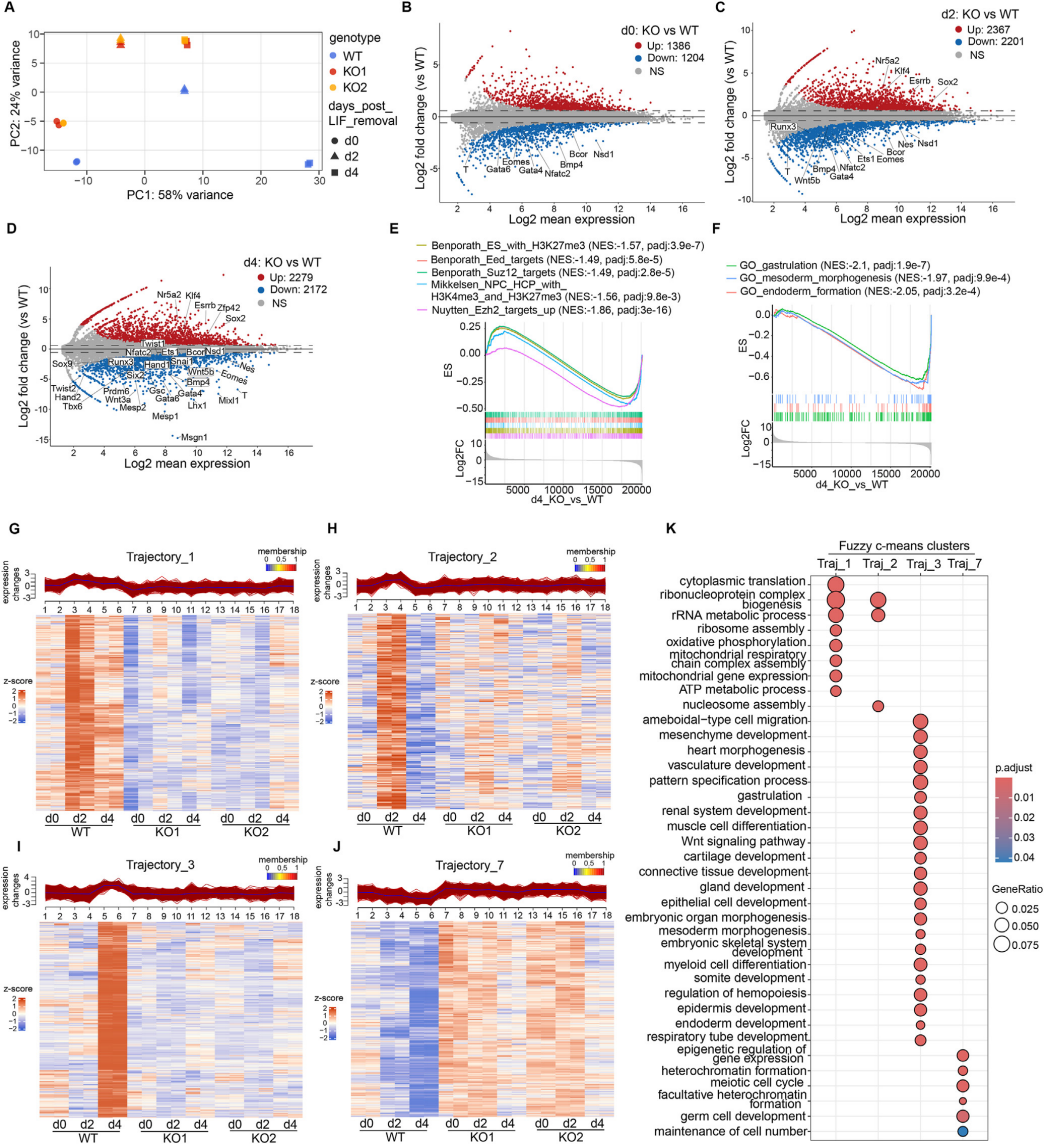
**Nsd1 facilitates transcriptional induction of the poised developmental genes during mESC differentiation.***A*, PCA of RNA-seq profiles, collected at three different time points (day 0, 2, and 4) after withdrawal of LIF and MEFs to induce the monolayer differentiation of the E14 mESCs, either Nsd1-WT or Nsd1-KO (n = 2 replicates per genotype at each time point). *B*–*D*, MA plots displaying the differentially expressed genes (DEGs) identified in Nsd1-KO *versus* WT cells at day 0 (*B*), day 2 (*C*), and day 4 (*D*), respectively, following differentiation. The upregulated and downregulated DEGs, called with a significance cutoff of padj value less than 0.01 and the absolute value of log2 converted value of fold-change (FC) more than 0.58, are presented by *red* and *blue dots*, respectively, with key meso-endoderm specifying TF genes and stemness-related genes labeled and highlighted. *E* and *F*, GSEA showing the enrichment of the indicated gene sets against a ranked RNA-seq gene list (KO *versus* WT at day 4 post differentiation). NES, normalized enrichment score. *p* values were determined by an empirical phenotype-based permutation test and BH-adjusted for gene set size and multiple hypotheses testing. *G* to *J*, Fuzzy c-means clustering of RNA-seq datasets identifies eight clusters (named as Trajectory_1 to Trajectory_8) with distinct trajectory patterns. Traj_1 (*G*), Traj_2 (*H*), Traj_3 (*I*), and Traj_7 (*J*) are shown as *line plots* (*top*) and heatmaps (*bottom*) of z-score expression. *Black lines* in the line plots are cluster centroid; genes are colored by the degree of cluster membership; genes with max_membership > 0.9 are plotted. *K*, dot plot comparing GO terms enriched in the genes of Traj_1 (n = 1034), Traj_2 (n = 4 56), Traj_3 (n = 1416), and Traj_7 (n = 1186). Dots are differentially colored and sized based on their corresponding BH-adjusted *p* values and gene ratio values, respectively. *p* values were calculated by hypergeometric test and BH-adjusted for multiple comparisons. GO, gene ontology; GSEA, Gene Set Enrichment Analysis; LIF, leukemia inhibitory factor; MEF, mouse embryonic fibroblast; mESC, mouse embryonic stem cell; NSD1, nuclear receptor-binding SET domain-containing protein 1; PCA, principal component analysis; RNA-seq, RNA sequencing; TF, transcription factor.

To systematically define what molecular pathways are perturbed by Nsd1 loss during mESC differentiation, we performed a time-course bulk cell RNA sequencing (RNA-seq) analysis of the Nsd1-WT *versus* Nsd1-KO cells at day 0, 2 and 4 after the monolayer cell differentiation. Principal component (PC) analysis (PCA) revealed that WT cells, based on their differentiation time points, were fully separated and aligned along the PC1 axis from the left to right, which represents a trajectory of normal mESC differentiation (Fig. 3*A*). In contrast, their Nsd1-KO cell counterparts displayed a rather limited separation in the PCA plot following 4 days’ differentiation, suggesting that Nsd1 KO results in a delay of differentiation progression and a retention of the stem cell state (Fig. 3*A*). We next analyzed the differentially expressed genes (DEGs) in these Nsd1-KO *versus* Nsd1-WT cells at each time point (Fig. 3, *B*–*D*; Tables S2–S4). As expected, more genes became differentially expressed following the time course of differentiation (*i.e.*, day 4 or 2 vs. day 0), presumably due to increased difference in cell states between the two (Fig. 3, *B*–*D*). As differentiation progresses, an increasing number of bivalent genes (27), particularly those developmental TFs that govern formation of the primary germ layers and diverse pathways of organ morphogenesis, failed to be efficiently induced in the Nsd1-KO cells, when compared to the WT controls (highlighted in Figs. 3, *B*–*D*, and S3*A*). These above TFs are enriched with those known to control gastrulation and meso-endoderm formation (such as T, Gsc, Eomes, Gata4, Mixl1, Msgn1, and Snai1) (28, 29, 30), along with others reported to direct the organogenesis of various mesoderm-derived tissues such as heart (*e.g.*, Mesp1, Mesp2, Gata6, and Tbx5) (31, 32), kidney (*e.g.*, Lhx1) (33), hematopoietic (*e.g.*, Runx3 and Nfatc2) (34, 35) and skeletal system (*e.g.*, Hand1, Hand2, Twist1, Twist2, Sox9, and Six2) (36). It is also noteworthy to mention that the basal expression levels of certain key developmental TFs (such as T and Gata4) already decreased at the steady-state ESC stage upon Nsd1 ablation (Fig. 3*B*), highlighting a role of Nsd1 in priming activation of the developmental TF genes. Meanwhile, we observed that several master stemness TFs (such as Sox2 and Nr5a2), which are normally silenced upon mESC differentiation to enable the pluripotency exit, were aberrantly retained at higher levels in Nsd1-KO *versus* WT cells (as highlighted in Fig. 3, *B*–*D*).

We next performed Gene Set Enrichment Analysis to unbiasedly characterize the gene pathway perturbations induced by Nsd1 KO at each time point. Genes known to be bound by PRC2 or H3K27me3, or cobound by H3K4me3 and H3K27me3, tend to be expressed at lower levels in Nsd1-KO cells relative to WT controls, which is persistent throughout the differentiation trajectory (Figs. 3*E*, and S3, *B* and *D*). This observation indicated that, upon Nsd1 loss, the differentiation blockade likely stems from a gain or expansion of H3K27me3. Pathways uniquely downregulated in Nsd1-KO *versus* WT cells at day 0 and day 2 post differentiation were related to ribosomal biogenesis, translation, and mitochondrial electron transport chain, which likely contributes to the full-blown defective differentiation phenotype observed at day 4 (Fig. S3, *C* and *E*). Finally, as differentiation advances, a growing number of development-related gene pathways became compromised in Nsd1-KO cells, which in turn maintained an aberrant response to LIF (Figs. 3*F* and S3, *F*–*I*).

To further dissect how normal differentiation transitions were perturbed after Nsd1 KO, we conducted trajectory analysis of gene expression changes for each sample from day 0 to day 4 following differentiation, and grouped DEGs into 8 clusters based on the distinct trajectory patterns (named as Trajectory_1 to Trajectory_8) by using Fuzzy c-means clustering analysis (Figs. 3, *G*–*J*, S3, *J*–*M*, Table S5). Among these trajectory clusters, Trajectory_1 (Traj_1), Traj_2, Traj_3, and Traj_7 were most informative. Specifically, genes of Traj_1 and Traj_2 participate in ribosomal biogenesis and oxidative phosphorylation (*e.g.*, ribosomal protein-coding genes such as Rpl13 and Rps21, and mitochondrial respiratory chain-coding genes such as Cox5b and Ndufa1), which exhibited initial upregulation at day 2 followed by downregulation at day 4 post differentiation of WT mESCs, whereas their levels remained constantly low in the Nsd1-KO cells (Fig. 3, *G*, *H* and *K*). Traj_3 contains genes that are related to embryonic development and remarkably induced at day 4 post differentiation of WT mESCs (*e.g.*, T, Gata4, Eomes, Hand1, Lhx1, Mixl1, and Msgn1); in contrast, these genes failed to do so in Nsd1-KO cells (Fig. 3, *I* and *K*). Genes of Traj_7 were progressively downregulated over time in WT mESCs; however, these genes displayed the markedly higher levels in Nsd1-KO cells across the time points, with only a marginal reduction seen at day 4 post differentiation (Fig. 3*J*). Genes of Traj_7 (*e.g.*, Sox2, Setdb1, and Trim28) contribute to heterochromatin formation, epigenetic regulation, and germ cell development (Fig. 3*K*). Finally, genes of Traj_4 and Traj_5 exhibit either downregulation or upregulation over time in a comparable manner between WT and Nsd1-KO cells (Fig. S3, *J*, *K* and *N*). Traj_6 and Traj_8 include genes either downregulated or upregulated to a greater extent in Nsd1-KO than WT cells (Fig. S3, *L*–*N*).

Collectively, these data indicate that Nsd1 is essential for the temporary boost of cellular machineries involved in protein translation and energy production at earlier stages of the *in vitro* mESC differentiation, as well as the activation of bivalent developmental genes, particularly those meso-endoderm cell lineage-specifying TF genes, at the later stages of differentiation.

### Nsd1-catalyzed H3K36me2 is essential for induction of bivalent gene expression and appropriate differentiation of mESCs

**Figure 4:**
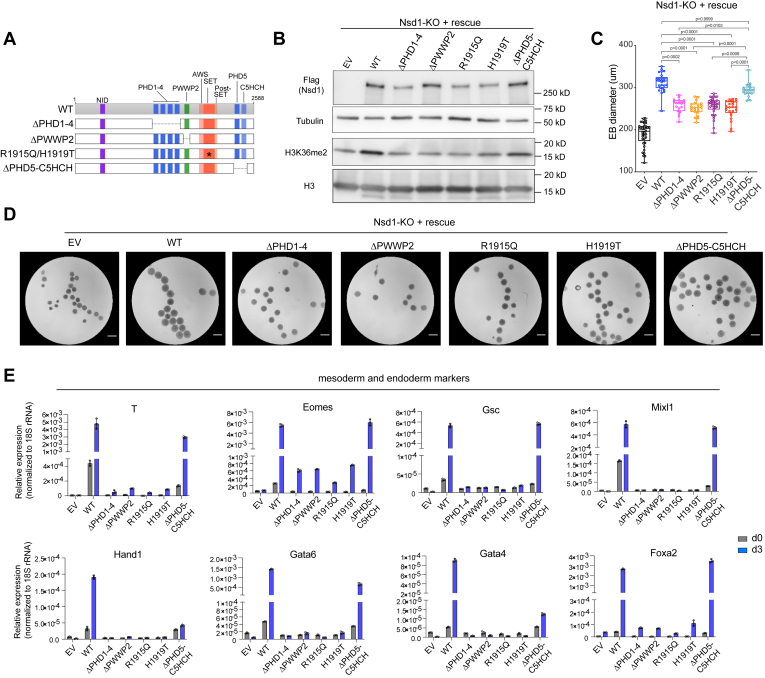
**Nsd1-catalyzed H3K36me2 is essential for induction of bivalent gene expression and appropriate differentiation of mESCs.***A*, a schematic showing the protein domain architecture of Nsd1 and different mutant constructs. Δ depicts domain deletion. *B*, immunoblotting for H3K36me2 and 3×Flag-tagged Nsd1, either WT or the indicated mutant stably transduced into the Nsd1-KO mESCs. EV, empty vector. *C*–*E*, distribution of diameters of EBs (*C*, n = 37, 26, 19, 22, 35, 24, and 25 from *left* to *right*), representative EB images 3 days post differentiation (*D*; the scale bar represents 400 um), and RT-qPCR analysis of the indicated genes (*E*) before or after a 3-day differentiation of Nsd1-KO mESCs with the stably transduced WT or mutant Nsd1. RT-qPCR signals from three independent experiments were normalized to those of 18S rRNA and presented as mean ± SD. Data in panel *C* were tested for normal distribution using the Kolmogorov–Smirnov test. Outliers in panel *C* were identified using the ROUT method. *p* values in panel *C* were determined using the Kruskal–Wallis test (*p* < 0.0001) followed by Dunn’s multiple comparisons test. EB, embryoid body; mESC, mouse embryonic stem cell; RT-qPCR, reverse transcription followed by quantitative PCR.

We next sought to determine which protein modules in Nsd1 are required for bivalent gene activation and thus the appropriate mESC differentiation. Toward this end, we used the Nsd1-KO mESCs and conducted Nsd1 rescue experiments with an exogenously introduced Nsd1, either WT or the mutant carrying the domain deletion or enzymatic-dead point mutation (R1915Q or H1919T in the catalytic SET domain) (Fig. 4*A*). As expected, introduction of WT Nsd1, but not the SET mutant, restored the global level of H3K36me2 (Fig. 4*B*). Intriguingly, in-frame deletion of either PHD1-4 modules or the PWWP2 domain in Nsd1 also abrogated the H3K36me2 deposition to a degree like what was seen with the SET mutant, whereas the tandem PHD5-C5HCH domains are largely dispensable for H3K36me2 deposition (Fig. 4*B*). Also, it is worth noting that the protein stability of Nsd1 depends on the intact PHD1-4 and SET domains; thus, the defects seen with the mutation of PHD1-4 and SET domains could be due to the Nsd1 protein instability or loss-of-function of these protein modules or both.

We next evaluated the capability of different Nsd1 constructs in rescuing defective EB formation caused by Nsd1 KO. At day 3 post differentiation, EBs differed in their sizes in a manner reminiscent of the extent of H3K36me2 restoration (Fig. 4, *C* and *D*). Specifically, WT EBs manifested the biggest sizes, followed by EBs with ΔPHD5-C5HCH, and then the four H3K36me2-low mutants (ΔPHD1-4, ΔPWWP2, R1915Q, and H1919T). Of particular interest, the four H3K36me2-low mutant EBs were still bigger than Nsd1-KO controls despite bearing comparable H3K36me2 levels. This implies that both H3K36me2-catalytic activity and putative catalytic-independent adaptor functions of Nsd1 contribute to appropriate differentiation of mESCs.

In align with the above observed difference in EB sizes, the development-accompanied induction of meso-endoderm-specifying TF genes was potently recovered by WT Nsd1, and to a slightly lesser extent by ΔPHD5-C5HCH mutant as well, following their rescued expression in Nsd1-KO cells (Fig. 4*E*). In contrast, the four H3K36me2-low mutants (ΔPHD1-4, ΔPWWP2, R1915Q, and H1919T) only modestly restored the expression of some lineage-specifying TF genes (such as T, Eomes, and Foxa2), but not other tested key TFs (such as Gsc, Mixl1, Hand1, Gata6, and Gata4) (Fig. 4*E*). In parallel, stemness TFs were silenced to the greatest degree in EBs with WT Nsd1, followed by those with ΔPHD5-C5HCH and then the four H3K36me2-low mutants (Fig. S4). Finally, we also noted that the basal expression levels of the above bivalent TF genes before differentiation (at day 0) were often elevated among WT Nsd1-rescued cells and to a lesser degree ΔPHD5-C5HCH mutant rescued cells, underlining the necessity of Nsd1 in safeguarding key developmental genes in a primed state (Fig. 4*E*).

Altogether, these results suggest that the Nsd1-mediated deposition of H3K36me2 relies on intact PHD1-4, PWWP2 and SET, but not the ΔPHD5-C5HCH tandem domains. Both catalytic activity and putative catalytic-independent functions of Nsd1 may aid in appropriate and timely induction of bivalent gene expression and mESC differentiation.

### Nsd1 safeguards the developmentally critical enhancers in a poised state by antagonizing H3K27me3 and facilitating H3K27ac

**Figure 5:**
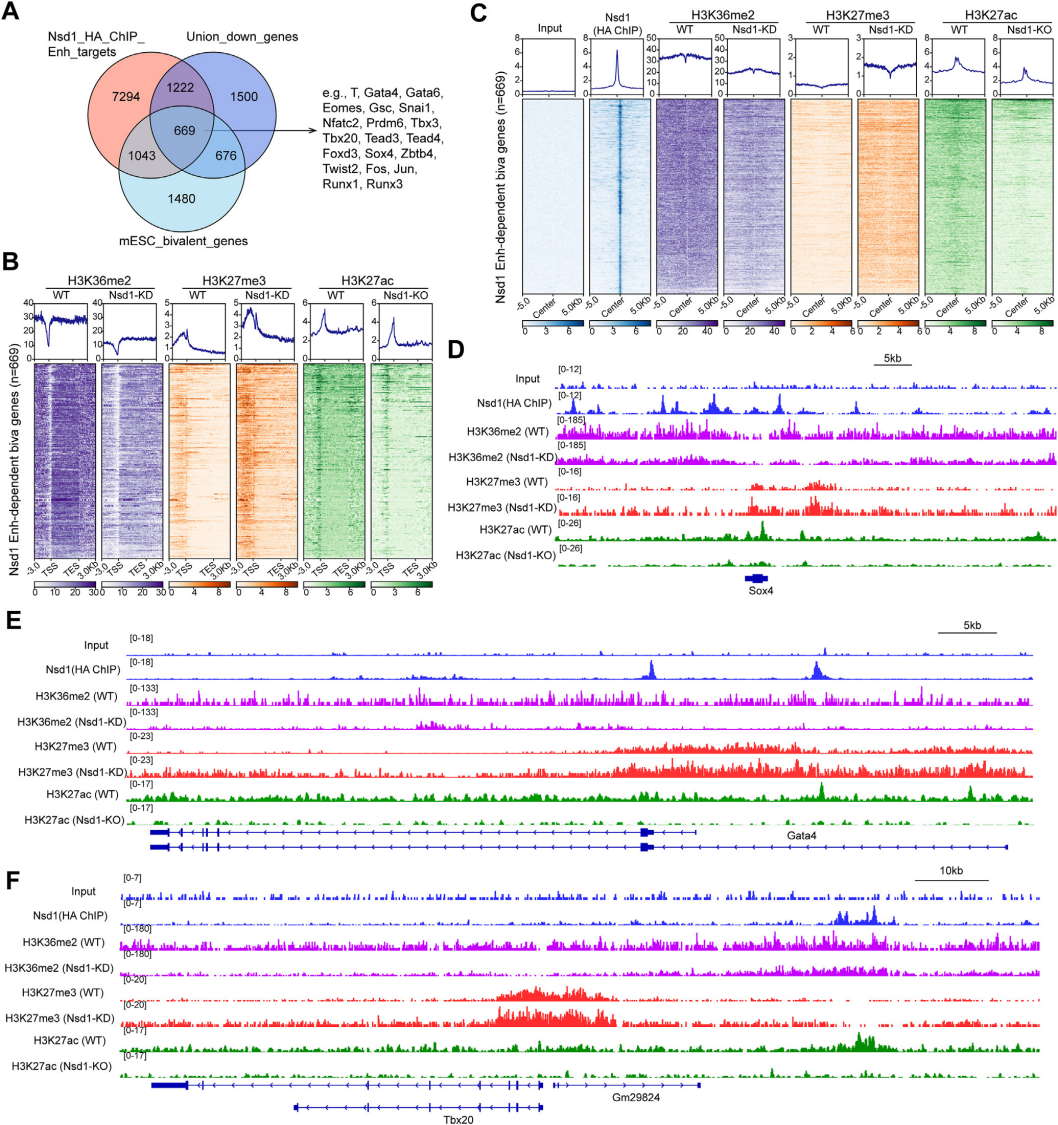
**Nsd1 safeguards the developmentally crucial gene enhancers in a poised state by antagonizing H3K27me3 and facilitating H3K27ac.***A*, Venn diagram using genes associated with the Nsd1-12×HA-bound enhancers (Nsd1_HA_ChIP_Enh_targets) in mESCs, genes downregulated in the Nsd1-KO versus WT cells across any of the three differentiation time points (day 0, 2, and 4; Union_down_genes), and the known genes exhibiting a bivalent domain feature in mESCs. Examples of such Nsd1-dependent bivalent target genes are highlighted on the *right*. *B* and *C*, average signal profiles and heatmaps displaying the indicated ChIP-seq signals at Nsd1-dependent bivalent genes (n = 669, defined in the panel *A*), either along the gene transcription units (*B*, 3kb beyond a gene-coding region) or over 10kb regions centered around the enhancer-bound Nsd1-12×HA ChIP-seq peaks at these genes (*C*) in WT or Nsd1-deficient mESCs. *D* to *F*, IGV views of the indicated RPGC-normalized ChIP-seq signals at Sox4 (*D*), Gata4 (*E*), and Tbx20 (*F*) loci in WT or Nsd1-deficient mESCs. ChIP-seq, chromatin immunoprecipitation sequencing; HA, hemagglutinin; IGV, Integrative Genomics Viewer; mESC, mouse embryonic stem cell; NSD1, nuclear receptor-binding SET domain-containing protein 1; RPGC, reads per genome coverage.

H3K36me2 is known to counteract the spreading of H3K27me3 (14, 37). We next queried whether loss of the Nsd1-catalyzed H3K36me2 leads to unrestrained H3K27me3 expansion and consequently, H3K27ac reduction, thereby shifting the balance of bivalent enhancers from a poised to a more stably repressed chromatin state. To test this idea, we first identified a list of bivalent genes that are directly bound by Nsd1 at their enhancers in the pluripotent mESC state and manifest a compromised basal expression and/or failure in induction upon differentiation (*i.e.*, those Nsd1-targeted genes exhibiting downregulation in the Nsd1-KO *versus* WT cells across different time points). This gene list (n = 669) harbors key meso-endoderm specifying TFs such as T, Gata4, Sox4, Snai1, Nfatc2, and Tbx20 (Fig. 5*A*). Leveraging on the published histone modification ChIP-seq datasets of WT and Nsd1 knockdown (KD)/KO mESCs (14, 38), we plotted the read tag densities of H3K36me2, H3K27me3, and H3K27ac at the gene bodies (TSS to TES) and flanking regions (3kb from each end) of the Nsd1-regulated bivalent genes (Fig. 5, *B*, *D*–*F*). Notably, Nsd1 loss-induced H3K36me2 reduction occurs uniformly throughout the plotted genomic regions, which is accompanied by dramatic elevation of H3K27me3 and shrink of H3K27ac, pointing to a pivotal role of Nsd1 in safeguarding developmental genes in a poised state that is primed for rapid induction upon receiving differentiation cues. Likewise, we observed a similar pattern of histone modification reprogramming when anchoring the plotting regions around Nsd1 peaks at the above bivalent gene enhancers in the Nsd1-deficient *versus* WT mESCs (Figs. 5*C* and S5, *A* and *B*).

Together, these data support a notion that Nsd1 directly binds and protects the bivalent developmental enhancers from excessive deposition of H3K27me3 in mESCs, thereby maintaining them in a poised state that is more responsive for differentiation-associated induction of transcription.

### Nsd1 continuously resides at the key developmental genes during the mESC differentiation

**Figure 6:**
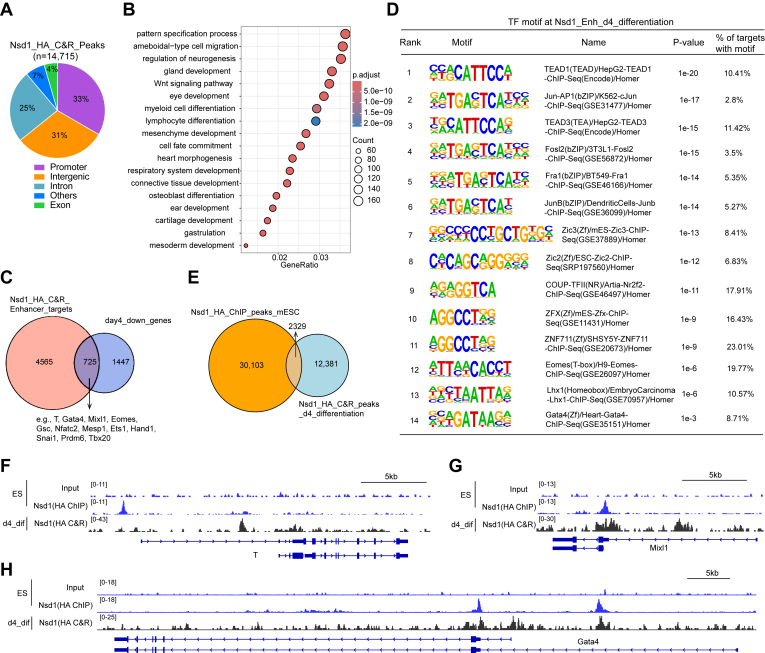
**Nsd1 continuously resides at key developmental genes during the stem cell differentiation.***A*, pie chart showing the genomic feature distribution of the Nsd1-12×HA CUT&RUN peaks at day 4 after monolayer differentiation of mESCs. Other features include 5′UTR, 3′UTR, ncRNA, and TTS. *B*, dot plot showing GO terms enriched among target genes of Nsd1-bound putative enhancer peaks (based on Nsd1-12×HA CUT&RUN, including intergenic and intronic regions) at day 4 after monolayer differentiation. *p* values were calculated by hypergeometric test and BH-adjusted for multiple comparisons. *C*, Venn diagram showing overlap between target genes associated with the Nsd1-bound enhancers and genes downregulated in Nsd1-KO *versus* WT cells at day 4 after mESC differentiation. *D*, summary of the top TF motifs enriched at Nsd1-bound enhancer peaks in cells after 4 days’ mESC differentiation. Motif enrichment was statistically determined by ZOOPS scoring (zero or one occurrence per sequence) coupled with the hypergeometric enrichment calculations. *E*, Venn diagram showing the overlap between Nsd1-12×HA peaks in un-differentiated mESCs and in cells after 4 days’ mESC differentiation. *F*–*H*, IGV views of Nsd1 binding (RPGC-normalized) at the indicated gene in mESCs and in differentiating cells (day 4 after mESC differentiation). CUT&RUN, Cleavage Under Targets and Release Using Nuclease; GO, gene ontology; IGV, Integrative Genomics Viewer; mESC, mouse embryonic stem cell; NSD1, nuclear receptor-binding SET domain-containing protein 1; RPGC, reads per genome coverage; TF, transcription factor; TTS, transcription termination site.

To further investigate whether Nsd1 also acts to stimulate the bivalent gene transcription in the differentiated cells, we performed Cleavage Under Targets and Release Using Nuclease (CUT&RUN) assays with mESCs expressing endogenous 12×HA-tagged Nsd1 and anti-HA antibody at day 4 following the monolayer differentiation of mESCs. Genomic feature annotation showed the Nsd1 peaks in these differentiating cell populations to be primarily enriched at putative enhancer regions, such as intergenic and intronic regions, which account for approximately 56% of all Nsd1 peaks (Fig. 6*A*). GO enrichment analysis revealed that these Nsd1-bound enhancer peaks tend to regulate genes involved in cell differentiation processes (Fig. 6*B*). In agreement, a considerable fraction of genes showing downregulation in the Nsd1-KO *versus* WT cells at day 4 after differentiation were directly bound by Nsd1 at their putative enhancers (Fig. 6*C*). Again, these Nsd1-targeted genes include key meso-endoderm specifying TFs such as T, Gata4, Mixl1, and Hand1 (Fig. 6, *C*, *F*–*H*). When compared to the TF motifs enriched at Nsd1-targeted enhancers under the pluripotent mESC state (primarily the motifs of ZFX and ZNF711; Fig. 1*I*), we identified a different and more diverse set of TF motifs at their counterpart genomic sites in the differentiating cell populations, which included the motifs specific to the TEAD and AP-1 family TFs (Fig. 6*D*). The latter set of TFs associated with Nsd1-bound enhancer peaks is known to mediate organogenesis during embryonic development (39, 40). This suggests the existence of a dynamically evolving mechanism for mediating Nsd1’s chromatin recruitment, which also aligns with the observation that Nsd1 peaks are largely redistributed upon cell differentiation (Fig. 6*E*), even at the same target genes (Fig. 6, *F*–*H*). Therefore, Nsd1 is likely required for the proper induction and transcriptional activation of developmental genes throughout the course of embryogenesis, lineage specification, and cell differentiation, which awaits further investigation.

## Discussion

In this study, we employed the mESC *in vitro* differentiation as a model system to interrogate the roles for Nsd1 in the regulation of epigenomic, transcriptomic states, as well as the cell state transition. Our genome-wide profiling uncovered that Nsd1 predominantly binds the intergenic and intronic regions where its occupancy levels are positively correlated with the enhancer activation markers (such as H3K4me1 and H3K27ac), suggesting Nsd1 to be a putative coactivator of gene enhancers. Notably, only a small fraction of the Nsd1-bound enhancer elements carry the active enhancer features and these enhancers are associated with cell stemness and proliferative genes (*e.g.*, Nanog, Pou5f1, and ribosomal protein genes). Meanwhile, a majority of Nsd1-bound enhancers are linked to genes involving embryogenesis and development (*e.g.*, T and Gata4), which manifest a poised bivalent chromatin state in mESCs (25) and are comarked by low levels of H3K4me1 and H3K27ac as well as high level of H3K27me3, as opposed to high levels of H3K4me1 and H3K27ac as well as low level of H3K27me3 seen at active enhancer elements. Intriguingly, despite the high levels of Nsd1 binding at their enhancers, the master stemness genes (*e.g.*, Nanog and Pou5f1) retain the largely unaltered transcription after Nsd1 ablation, suggesting the existence of redundant mechanisms for ensuring mESCs’ self-renewal gene programs. This phenomenon also aligns well with previous observations that the Nsd1-deficient mESCs maintain a normal self-renewal and proliferation activity (14). In contrast, the genes downregulated upon Nsd1 KO in mESCs tend to gain H3K27me3, many of which are involved in the developmental processes. Together, these observations highlight Nsd1 as a critical regulator of the developmental enhancers with the bivalent chromatin feature. The importance of Nsd1 in (epi)genomic regulation is also supported by its recurrent mutations and deregulation in a wide range of human diseases such as Sotos syndrome, Weaver syndrome, and pediatric leukemias (4, 5, 6, 7, 9, 10, 13, 41).

Compared to the WT counterpart, Nsd1-KO mESCs exhibit the profound defects in cell differentiation and impairment in the induction of development genes, particularly, a suite of master TFs (*e.g.*, T, Gata4, Mixl1, Snai1, and Nfatc2), which are known to act to specify the meso-endoderm lineages such as those in the cardiovascular and skeletal systems. Importantly, the above Nsd1 KO-induced defects largely recapitulate anomalies seen in the Sotos syndrome patients (such as overgrowth, advanced bone age, and congenital heart defects), supporting the relevance of our model system for dissecting the molecular mechanisms underlying pathogenesis of this syndrome. We also systematically assessed the roles of various protein modules in Nsd1 by performing rescue in Nsd1-KO mESCs with either WT Nsd1 or a mutant lacking the individual domain(s) or carrying the function-disrupting point mutation. In contrast to the largely dispensable PHD5-C5HCH domains, the PHD1-4 and PWWP2 domains, as well as the catalytic SET domain, are strictly required for global restoration of H3K36me2 and appropriate upregulation of developmental gene programs upon cell differentiation. Given that prior reports have shown the tandem PHD5-C5HCH domains to be critical for the association with target chromatin or partner factors and for leukemogenic capacity of a Nup98-Nsd1 fusion protein (13, 19), the functional significance of PHD5-C5HCH is likely to be context-dependent. Of note, the Nsd1 mutants incapable of depositing H3K36me2 efficiently also failed to rescue the basal expression or the efficient activation of bivalent developmental genes upon differentiation induction. Epigenomic analysis revealed that the loss of Nsd1 led to expansion of H3K27me3 and concomitant reduction of H3K27ac at the Nsd1-dependent bivalent genes, consistent to previous reports (13, 14, 15, 16, 17). Regarding the mechanism underlying Nsd1/H3K36me2-directed antagonism of H3K27me3, the previous studies have reported that H3K36me2 (or H3K36me3) does not affect PRC2's binding to the nucleosome and instead, it allosterically inhibits PRC2's catalytic activity by impairing the engagement between the catalytic center of EZH2 and the H3K27 substrate (42, 43, 44). Together, Nsd1 and the catalyzed H3K36me2 marks provide a safeguard mechanism for potentiating the cell differentiation-related induction of developmental genes, at least partly through antagonizing excessive deposition of H3K27me3.

Unlike SETD2 which binds the elongating RNA Pol II and cotranscriptionally deposits H3K36me3 along the body regions of actively transcribed genes (11, 45), how exactly Nsd1 establishes the broad domains of H3K36me2, as well as whether this process is coupled with the RNA Pol II activity, remains to be further investigated. Sun *et al.* recently showed that Nsd1 can facilitate the release of paused RNA Pol II at gene promoters, as evidenced by the decreased occupancy of Ser2-phosphorylated RNA Pol II and the reduced production of enhancer RNAs upon acute degradation of Nsd1 (21); and such a function of Nsd1 was linked to the recruitment of cellular factors promoting transcription elongation such as SPT5, PAF1, and SPT6 (21). Additionally, other studies suggest that H3K36me2 is likely to promote productive elongation of RNA Pol II by recruiting the H3K36me2-specific reader proteins (notably, LEDGF and HDGF2), which can in turn relieve the nucleosome-induced barrier to transcription (46, 47).

Many family members of the PHD fingers and PWWP domains were previously shown to bind the specific histone modification (20, 48, 49, 50). However, the functions of various PHD and PWWP modules within Nsd1 remain elusive to date. Due to a requirement of these PHD and PWWP modules for Nsd1’s protein stability in cells, their exact contribution to Nsd1-elicited (epi)genomic regulation such as H3K36me2 deposition can be hard to dissect. A recent report showed that the H3K18ac-modified mononucleosomes can efficiently pull down a fragment of Nsd1 protein spanning from PHD1-4, PWWP2 to SET domains from the cell lysate, indicative of a mechanism used by Nsd1 in the chromatin association (21). However, given that Nsd1’s genomic binding sites mainly reside at the bivalent enhancer regions, which are largely devoid of histone acetylation, we speculate that other mechanisms exist to direct Nsd1’s recruitment to its genomic target sites. For instance, Nsd1 was initially discovered due to a direct protein-protein interaction with the nuclear receptor family of TFs (18). In consistence, our unbiased motif search analysis identified the ZFX and ZNF711 consensus motifs to be most enriched among the Nsd1-bound enhancers in mESCs, suggesting a TF-dependent targeting mechanism.

It is worth noting that, despite smaller than their WT counterpart, the EBs with restored expression of the H3K36me2-deposition-defective Nsd1 mutant are still significantly bigger than the Nsd1-KO controls, indicative of a catalytic-independent function of Nsd1 during the mESC differentiation. In agreement, we observed a mild but consistent induction of certain meso-endoderm lineage-specification genes (such as T, Eomes, and Foxa2) among EBs reexpressed with the Nsd1 mutants incapable of H3K36me2 catalysis, when compared to their Nsd1-null controls. Likewise, a recent work documented that the Nsd1 KO-induced perturbation of nascent mRNA transcription can be equally rescued by WT and a catalytic-dead Nsd1 mutant, although the underlying mechanism remains to be determined (21). Therefore, the multifaceted activities of Nsd1, both catalytically dependent and catalytically independent, mediate Nsd1-related (epi)genomic regulation during development and cell differentiation. Our analysis of the Nsd1 interactome (unpublished) captured a wide range of chromatin-modulatory enzymes and regulators, underlining the extensive crosstalk between Nsd1 and other chromatin factors. Lastly and similar to other chromatin regulators already studied in the literature (*e.g.*, ARID1A, HP1, CBX2, UTX, MED1, and BRD4) (51, 52, 53, 54), the interdomain protein region of Nsd1 is largely disordered and is likely to mediate multivalent interactions that can facilitate the biomolecular condensation of Nsd1 and associated partner complexes, which may in turn fine-tune the target gene transcription.

Lastly, our results suggest Nsd1 to be broadly involved in the specification of multiple cell lineages during development. Given that different cell lineages differ significantly in their epigenetic landscape (such as Nsd1-deposited H3K36me2 *versus* PRC2-deposited H3K27me3/2) and in Nsd1’s genomic targeting mechanisms (such as distinct partner TFs, among other cellular contexts), Nsd1 is likely to function in a cell type-specific manner. In agreement with such a notion, NSD1 was suggested to act as both an oncogene and a tumor suppressor gene in different cancers (11, 13, 46, 55, 56). For instance, inactivation and frameshift mutations of NSD1 are frequent in patients with head and neck squamous cell carcinoma, which were reported to manifest an immune-cold tumor microenvironment partly due to the elevated H3K27me3 levels at key T cell-recruiting chemokines, CXCL9 and CXCL10 (55, 56). By contrast, the gain-of-function mutation of NSD1 and formation of the NUP98-NSD1 fusion due to its in-frame fusion to NUP98, a factor carrying capabilities for condensation (57, 58) and coactivator recruitment (59, 60), points to an oncogenic role of NSD1 in driving aberrant expression of HOX genes in patients with acute myeloid leukemia (13, 41). Furthermore, the unbalanced activation of NSD1 and expansion of H3K36me2 domains are suggested to mediate the oncogenic effect of the so-called oncohistone mutant, H3K27M, in diffuse intrinsic pontine glioma (46). Further studies are warranted to elucidate the detailed mechanisms underlying the cell type-specific functions of NSD1. A better understanding of NSD1-directed (epi)genomic regulation shall help to develop new therapies for the treatment of various human diseases caused by NSD1 dysregulation.

## Experimental procedures

### Cell culture

The E14 mESCs were obtained from American Type Culture Collection and cultured in the Dulbecco's modified Eagle's base medium supplemented with 15% of heat-inactivated fetal bovine serum, 1% of nonessential amino acid, 0.1 mM β-mercaptoethanol, 1% of antibiotics, and 1000 U/ml recombinant mouse leukemia inhibitory factor (LIF, Sigma-Aldrich, cat# ESG1107). E14 mESCs were grown either on top of a feeder layer of mitotically inactivated mouse embryonic fibroblasts (MEFs, Thermo Fisher Scientific, cat# A34963) in tissue culture (TC)-treated dishes, or in the TC plate precoated by 0.1% gelatin. Cells were monitored daily under light microscopy.

### Plasmids

CRISPR RNA (crRNA) targeting a genomic region proximal to Nsd1’s stop codon was designed by using the online tool CHOPCHOP. The crRNAs were synthesized as two complementary single-stranded oligonucleotides, then annealed and cloned into the BbsI enzyme-digested pSpCas9(BB)-2A-GFP plasmid (PX458; Addgene cat# 48138) using the NEB Quick Ligase. Two 700bp-long sgRNA-resistant homologous DNA sequences flanking the stop codon of Nsd1 gene were synthesized as the dsDNA gene fragments, followed by ligation into the flanking sides (BsaI and BbsI sites) of 3xFlag-P2A-NeoR cassette in the pFETCH donor vector (Addgene cat# 63934) by using a Gibson Assembly method as described before (61, 62). For KI of 12×HA tag in-frame to the Nsd1 gene locus, the original 3xFlag-P2A-NeoR cassette in the pFETCH donor plasmid was replaced by an AviTag-12×HA-P2A-eGFP cassette through subcloning of the synthesized cassette to the restriction enzyme sites (BstZ17I and BstBI) of the plasmid. Nsd1 rescue plasmids were generated by cloning either full-length WT mouse Nsd1 complementary DNA (cDNA) (encoding 2588 amino acids, kindly provided by P. Chambon, Strasbourg, France) or domain-deleted/mutated Nsd1 cDNA into the PB-EF1a-IRES-NeoR vector (PiggyBac Transposon system, SBI cat# PB533A-2) by the Gibson Assembly method. All plasmids were verified by direct Sanger sequencing before use.

### CRISPR-Cas9-based editing of endogenous Nsd1 gene in mESCs

A 3xFlag-P2A-NeoR cassette was introduced in-frame to the C terminus of endogenous Nsd1 gene as previously described (62). Briefly, E14 cells were cotransfected with the PX458/pSpCas9(BB)-2A-GFP containing Cas9 and a Nsd1 C-terminus-targeting sgRNA, as well as the homology donor-containing pFETCH plasmids (for plasmid construction, see above), in a 1:2 M ratio by using PEI (Polysciences cat# 24765), followed by drug selection with 1 mg/ml G418 (Thermo Fisher Scientific, cat# 10131035) for 1 week, starting at 72 h post transfection, to enrich for edited cells. The PX458/pSpCas9(BB)-2A-GFP plasmid simultaneously expresses Cas9 and a guide RNA which targets a Nsd1 exon23 site 30 bp upstream of the stop codon. pFETCH-donor plasmid contains the 3×Flag-P2A-NeoR cassette flanked by two 700bp-long homology arms used to repair the DNA double-strand break through homologous recombination. Next, the drug-resistant cells were diluted in the culture medium and seeded into 96-well plates at a density of 0.5 cell per well to generate clonal lines for screening of biallelic KI clones. Two different primer pairs that span either 5′ or 3′ recombination sites were designed for detecting the desired recombination events at the Nsd1-edited alleles. In addition, a third primer pair targeting the two homology arms was used to distinguish homozygous *versus* heterozygous KI. All genotyping amplicons using genomic DNA as template were sequenced to ensure the accuracy of the editing. To tag endogenous Nsd1 C terminally with the AviTag-12×HA tag, E14 cells were electroporated with the ribonucleoprotein (RNP) complex containing recombinant Cas9 and a Nsd1-exon23-targeting sgRNA (IDT; for details, see below sections), together with a modified pFETCH-donor plasmid harboring the AviTag-12×HA-P2A-eGFP cassette using Nucleofector kit (Lonza, cat# VPH-1001), followed by sorting of GFP-positive single cells into 96-well plates and then screening for clones with biallelic editing as detailed above. For the clonal lines with homozygous KI based on genotyping and sequencing confirmation, we further verified them using the mRNA (RT-PCR of edited regions for direct Sanger sequencing) and using immunoblot with the antibody of Flag tag (Sigma-Aldrich, cat# F1804) or HA tag (abcam cat# ab9110). The used oligo sequences are provided in the Table S6.

### CRISPR/Cas9-mediated KO of endogenous Nsd1 gene in mESCs

Two different RNP complexes targeting the introns 4 and 5 of Nsd1, respectively, were simultaneously delivered into the above Nsd1-3×Flag E14 cells to delete exon 5 *via* electroporation following a protocol that we described before (62, 63). Specifically, crRNAs and the universal tracrRNA (transactivating crRNA) labeled with the ATTO 500 fluorescent dye at its 5′end (IDT cat# 1075927) were synthesized as RNA oligos and then annealed to form duplex, followed by incubation with the Cas9 enzyme (IDT cat# 1081060) in a 5 μl reaction volume containing 2.1 μl PBS, 1.2 μl 100 μM crRNA-tracrRNA duplex, and 1.7 μl 61 μM Cas9 at room temperature (RT) for 20 min to assemble the RNP complex. Two million E14 cells, suspended in 88 μl of the Nucleofector solution, were mixed with 2.5 μl of 96 μM Electroporation Enhancer (IDT cat# 1075915) and 5 μl of each RNP, then subject to electroporation using A-023 program on the Nucleofector II Device. Subsequently, 36 h after RNP introduction, the ATTO 500-positive single cells were sorted into 96-well plates. Two different genotyping primer pairs, located either outside or inside the two cutting sites, were utilized to screen for homozygous Nsd1-KO clones. The genotyping PCR products were sequenced to confirm the nonhomologous end joining and removal of exon 5. RT-qPCR and immunoblot using tag antibody were carried out to further validate the homozygous Nsd1 KO. The oligos used for Nsd1 KO are listed in the Table S6.

### *In vitro* differentiation of mESCs

To initiate monolayer differentiation, E14 cells were suspended in the above ESC cultivation medium without LIF and seeded to the gelatin-coated TC-treated dish at a density of 2 million cells per 10 cm dish at day 0. Cells were passaged every other day and collected at day 2 or 4 post differentiation for downstream studies such as RT-qPCR analysis of the cell stemness and lineage markers. In a second protocol, E14 mESCs were induced to form EBs using the hanging drop method (26). Briefly, after removing feeder MEFs, E14 cells were diluted to 10 cells/μL in the ESC medium without LIF and multichannel pipette was used to distribute drops in a volume of the 30 μl on the inside surface of bacterial Petri dish lid, followed by inverted culture for 3 days. Then, EBs were collected and cultured in the bacterial Petri dish as 3D spheroids with medium changed every other day. The EB images were taken under the light microscope and EB sizes were measured using ImageJ.

### Western blotting

Protein samples were separated by SDS-PAGE and then transferred to a polyvinylidene fluoride membrane. The membrane was first blocked with 5% nonfat dry milk in TBST buffer (20 mM Tris, pH 7.6, 150 mM NaCl, and 0.1% Tween 20) for 1 h at room temperature and subsequently incubated with primary antibody diluted in the blocking buffer overnight at 4 °C. The following primary antibodies were used: HA (Abcam, ab9110), Flag (Sigma-Aldrich, F1804), H3K36me2 (CST, 2901), H3K27me3 (Millipore, 07–449), H3 (CST, 9715), and Tubulin (CST, 2146). Primary antibodies were validated by the manufactures and the validating data were provided on the vendor websites. Furthermore, we used positive and negative controls to validate the antibody specificity in Western blotting or immunofluorescence. For histone antibodies, specificity was also extensively examined by many independent investigators in addition to the vendor (refer to http://www.histoneantibodies.com/). Following three washes with TBST, the membrane was incubated with goat anti-mouse IgG or goat anti-rabbit IgG conjugated to horseradish peroxidase at a dilution of 1:5000 in blocking buffer for 1 h at RT. After three washes with TBST, the blots were developed by Pierce ECL Western Blotting Substrate (Thermo Fisher Scientific) and imaged on a Bio-Rad ChemiDoc imager.

### RT-qPCR

Total RNAs were extracted and converted into cDNA using the iScript cDNA Synthesis Kit (Bio-Rad cat# 1708890). Quantitative PCR was performed in triplicate using the iTaq Universal SYBR Green Supermix (Bio-Rad cat# 1725124) on the QuantStudio 6 Flex Real-Time PCR System (Applied Biosystems). The quantitative PCR signal was normalized to an internal control (18S ribosomal RNA or Gapdh) using the ΔCT method, followed by a second normalization to signals of WT cell controls for fold change calculations in some cases. The primer sequences are provided in Table S6.

### Chromatin immunoprecipitation followed by deep sequencing

E14 mESCs carrying the Nsd1-3×Flag or Nsd1-12×HA alleles were used for Flag and HA ChIP-seq, respectively, as described before (64, 65). In brief, E14 single-cell suspension was subject to a dual cross-linking protocol, which includes the initial fixation in PBS with 2 mM disuccinimidyl glutarate (DSG; Thermo Fisher Scientific, cat# 20593) for 45 min at RT followed by fixation in PBS with 1% formaldehyde (Thermo Fisher Scientific, cat# 28908) for 10 min. The cross-linking was quenched by addition of 125 mM glycine for 5 min. The cross-linked cells were collected and sequentially lysed or washed in the previously described buffers LB1, LB2, and LB3, followed by sonication for 60 cycles on Bioruptor sonicator at a high energy setting with the 30s on and 30s off cycles (Diagenode cat# B01020001). The cleared sonication supernatant was then incubated with Flag (Sigma-Aldrich, cat# F1804) or HA (abcam cat# ab9110) antibody-bound Dynabeads (Thermo Fisher Scientific, cat# 11202D, 11204D) overnight at 4  °C. After sequential washes with low-salt buffer, high-salt buffer, LiCl buffer, and TE buffer, the ChIP product was eluted and subject to reverse crosslink overnight at 65 °C, followed by digestion with RNase (Roche cat# 11119915001) and Protease K (Roche cat# 03115828001) and then DNA recovery using the Qiagen PCR purification kit (Qiagen cat# 28106). The ChIP-seq libraries were generated using NEBNext Ultra II DNA Library Prep Kit (NEB cat# E7645L), which were sequenced on an Illumina Nextseq 500 Sequencer using Nextseq 500/550 High Output Kit v2.5.

### Cleavage Under Targets & Release Using Nuclease

CUT&RUN was performed following the EpiCypher CUTANA CUT&RUN Protocol as performed before (64). Briefly, one million of live cells (mESCs with Nsd1-12×HA) were washed and immobilized onto activated ConA magnetic beads (Bangs Laboratories cat# BP531) by incubation at RT for 10 min, followed by permeabilization and incubation with HA antibody (1:100 dilution) on a nutator overnight at 4 °C. On the next day, the cell-bead slurry was washed twice and incubated with pAG-MNase (1:20 dilution, EpiCypher cat# 15–1116) for 10 min at RT, followed by addition of CaCl_2_ and 2 h incubation at 4 °C for targeted chromatin cleavage by activated MNase. After chromatin digestion, the stop buffer was added and chromatin fragments released into supernatant were purified using the Monarch DNA Cleanup Kit (NEB cat# T1030) per manufacturer’s instruction. Additionally, 10 ng of purified DNA was subject to library preparation using the NEB Ultra II DNA Library Prep Kit (NEB cat# E7645). Libraries were loaded onto the Illumina NextSeq 550 System for sequencing in a pair-end 75 bp (PE-75) format.

### ChIP-seq and CUT&RUN data analysis

ChIP-seq data processing was performed as previously described with slight modifications (64). In brief, raw sequencing reads were aligned to mm10 mouse genome using STAR 2.7.10 b with splicing alignment disabled (--alignIntronMax 1 --alignEndsType EndToEnd) (66). Nonprimary aligned, duplicated reads and reads mapping to ENCODE blacklisted regions (67) were filtered out before downstream analysis. The MACS2 software 2.2.7.1 (68) was employed for peak calling using the default parameters (q < 0.05 as significance cutoff) and input as control. Peak annotation and TF motif enrichment analysis with peak summit bed file as input and a specified region size of 200 was carried out using the Homer 4.11 functions “annotatePeaks.pl” and “findMotifsGenome.pl”, respectively. Alignment bam files were also transformed into read coverage bigWig files using the deepTools 3.5.4 function “bamCoverage” with options [-bs 10 --centerReads -e 250] and normalized to reads per genome coverage (69). The resulting bigWig files were loaded into the Integrative Genomics Viewer software for visualization. K-means clustered heatmaps for reads per genome coverage-normalized ChIP-seq signals were produced using the deepTools “computeMatrix” and “plotHeatmap” functions, with the plotted regions sorted in a descending order based on the row means. CUT&RUN data were processed similarly as ChIP-seq data except that the peak significance cut off was set as q < 0.1.

### RNA sequencing

RNA-seq was performed as described before (64). Briefly, total RNAs were extracted using RNeasy Plus kit (Qiagen cat# 74136), followed by removal of residual DNAs using Turbo DNA-free kit (Thermo Fisher Scientific, cat# AM1907) to ensure purity of RNA sample. Poly-A selected mRNA-seq libraries were prepared by Novogene Inc and sequenced on Illumina NovaSeq 6000 (in a PE-150 bp format) to achieve a sequencing depth of >20 to 30 million read pairs per sample.

### RNA-seq analysis

The fastq files were trimmed using trim-galore 0.6.7 and then aligned to mm39 mouse genome using STAR 2.7.11a (66). Primary aligned, nonduplicated reads were supplied to featureCounts 2.0.6 for counting read fragments aligned to exons. Raw gene expression counts were further processed with DESeq2 1.42.0 (70). Low-count genes with an average count < 1 were filtered out before downstream analysis. DEGs were defined with a significance cutoff of adjusted P (padj) value less than 0.01 and the absolute log2 value of fold change (|log2FC|) over 0.58. PCA was performed on variance stabilizing transformation-normalized expression values for the top 500 most variable genes. MA plots were generated using the shrunken log2 fold changes, which removed noises associated with low-count genes. Fuzzy c-means clustering was performed to assign genes to distinct clusters displaying differential gene expression trajectories along differentiation. The optimal number of clusters was determined as the elbow point based on the minimum centroid distance versus cluster number plot. Gene Set Enrichment Analysis with the H, C2, and C5 gene set collections and GO pathway enrichment analysis with the biological process annotations was done using the R package clusterProfiler 4.10.0 (71) using a significance cutoff of Benjamini-Hochberg-adjusted *p* value less than 0.01.

### Statistics

Data in bar and line plots are presented as mean ± SD of three independent experiments unless otherwise noted. Statistical analyses were conducted using either GraphPad Prism 9 or the R statistical language, with the statistical methods specified in the corresponding figure legends. A *p* value or adjusted *p* value of less than 0.05 was considered significant unless otherwise noted.

## Data availability

All of the raw and processed sequencing data generated in this study have been submitted to the NCBI Gene Expression Omnibus (GEO; https://www.ncbi.nlm.nih.gov/geo/) under the accession numbers GSE271861 (ChIP-seq), GSE271862 (RNA-seq) and GSE271864 (Cut&Run). We also used the following previously published datasets: GSE47949 (H3K4me1, H3K4me3, H3K27ac, and H3K27me3 ChIP-seq in WT mESCs), GSE107773 (H3K36me2 and H3K27me3 spike-in ChIP-seq in WT and Nsd1-KD mESCs) and GSE186506 (H3K27ac ChIP-seq in WT and Nsd1-KO mESCs).

## Supporting information

This file contains supporting information (Figs. S1–S5, and legends to Tables S1–S6).

## Conflict of interest

The authors declare that they have no conflicts of interest with the contents of this article.

## Supporting information

2_Nsd1_SuppleInfor-JBC-revise_FINAL.pdf

Nsd1_Supplementary_Tables_1-6 with legend final.xlsx
