# Supplementary material for: The sotos syndrome gene Nsd1 safeguards developmental gene enhancers poised for transcription by maintaining the precise deposition of histone methylation[image]
Source: J Biol Chem. 2025 Mar 19;301(5):108423. doi: 10.1016/j.jbc.2025.108423 (PMC12033923; doi:10.1016/j.jbc.2025.108423)
Supplement: 2_Nsd1_SuppleInfor-JBC-revise_FINAL.pdf [file mmc1.pdf]

Supporting information

This file contains supporting information:
Legends to Supplementary Tables 1-6
Supplementary Figures 1-5 and legends

Table 1: Annotation of Nsd1-bound enhancer peaks, which are split into 3 k-means clusters using deepTools.

Table 2-4: Differentially expressed genes in Nsd1-KO vs WT cells at day 0 (**Table 2**), day 2 (**Table 3**), or day 4 (**Table 4**) of mouse ESC differentiation.

Table 5: Fuzzy c-means clustering analysis groups differentially expressed genes into 8 clusters showing distinct trajectory patterns along differentiation.

Table 6: oligo sequences used in the study.

Supplementary Figure 1

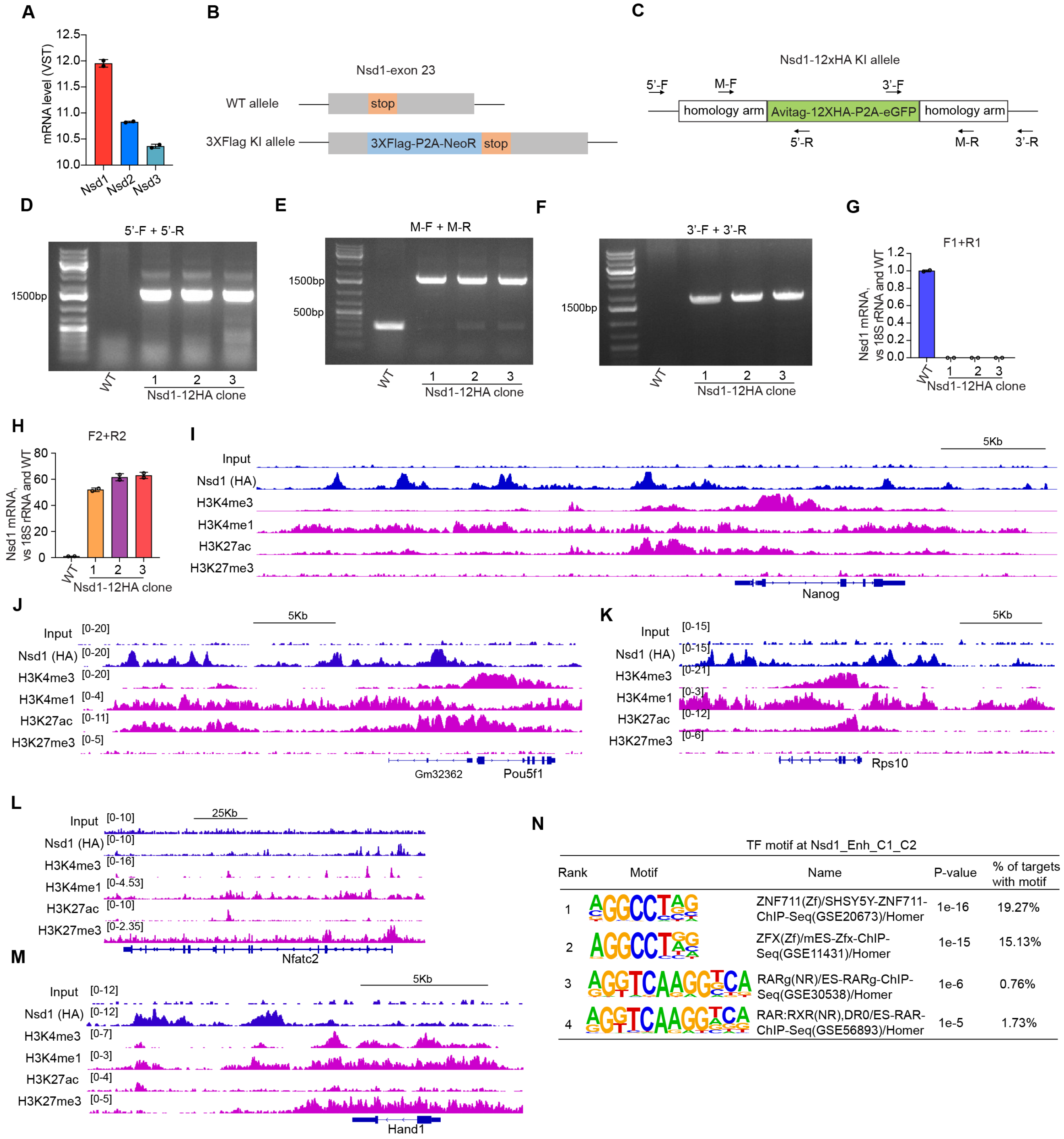

Supplementary Figure 1, Related to Figure 1.

(**A**) The mRNA level of Nsd1, Nsd2, and Nsd3 in mESCs, based on the variance stabilizing transformation (VST)-normalized read tags of RNA-seq profile data. Plotted is mean ± SD (n = 2 independent biological replicates).

(**B**) A schematic illustrating the in-frame knock-in (KI) of a 3×Flag-P2A-NeoR cassette to the C-terminus (exon 23 containing the stop codon) of endogenous Nsd1 gene allele.

(**C**) A schematic of primers used for genotyping of mouse ESCs with in-frame KI of a 12×HA-aviTag-P2A-eGFP cassette to the C-terminus of Nsd1.

(**D-F**) Agarose gel images of the PCR products from the Nsd1-12×HA KI allele genotyping. The locations of different primer sets, targeting either 5'-homology arm (5'-F and 5'-R), the KI cassette (M-F and M-R) or 3'-homology arm (3'-F and 3'-R), are shown in panel **C**. Un-tagged parental cells were used as a negative control.

(**G-H**) RT-qPCR analysis of Nsd1 mRNA in either parental or the three independent single-cell derived mESC clonal lines with the Nsd1-12×HA KI alleles. RT-qPCR signals, detected with the indicated primers, from two independent experiments were normalized to those of 18S rRNA and then to parental cells and then presented as mean ± SD. Primer locations are shown in main **Figure 1A**.

(**I-M**) IGV views of the indicated RPGC-normalized ChIP-seq signals at *Nanog* (**I**)*, Pou5f1* (**J**)*, Rps10* (**K**)*, Nfactc2* (**L**) *and Hand1* (**M**)*.*

(**N**) Summary of the top four most-enriched TF motifs at the Nsd1-bound C1+C2 enhancer peaks. Motif enrichment was statistically determined by ZOOPS scoring (zero or one occurrence per sequence) coupled with the hypergeometric enrichment calculations.

Supplementary Figure 2

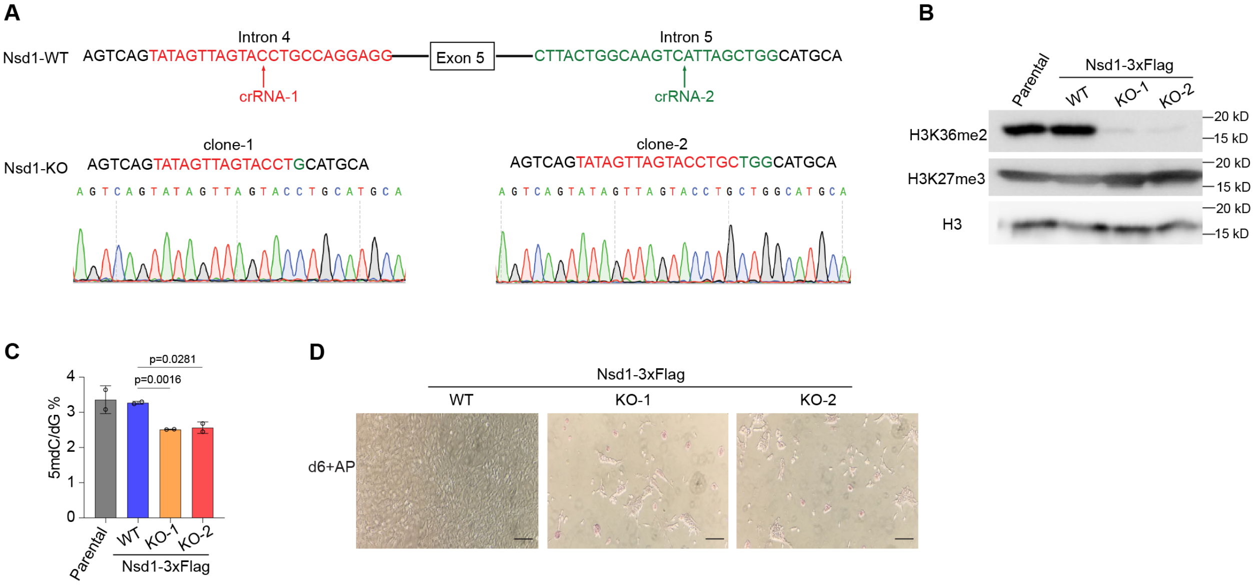

Supplementary Figure 2, Related to Figure 2.

(**A**) Design of CRISPR-Cas9-based deletion of the Nsd1 exon 5 (top; crRNA, CRISPR RNA) and results of Sanger sequencing using the genotyping PCR products (bottom), which confirmed homozygous deletion of the Nsd1 exon 5 in two independent clonal lines of Nsd1-KO mESCs.

(**B**) Immunoblotting for H3K36me2 and H3K27me3 in the parental E14 mESCs (lane 1) and those with the WT KI alleles of Nsd1_3×Flag (lane 2) or its KO (lanes 3-4 for two independent KO clonal lines).

(**C**) Mass spectrometry-based quantification of global DNA methylation level using total genomic DNA of the indicated mouse ESCs. The 5-methyl-deoxycytidine (5mdC) signal was normalized to deoxyguanosine (dG), an internal calibrant, and presented in y-axis as mean ± SD (n=2 independent biological replicates). The *P* values were calculated by two-sided Student's t test.

(**D**) Red-color alkaline phosphatase (AP) staining of Nsd1-WT and Nsd1-KO cells at day 6 after the monolayer differentiation. Scale bar, 500 um.

Supplementary Figure 3

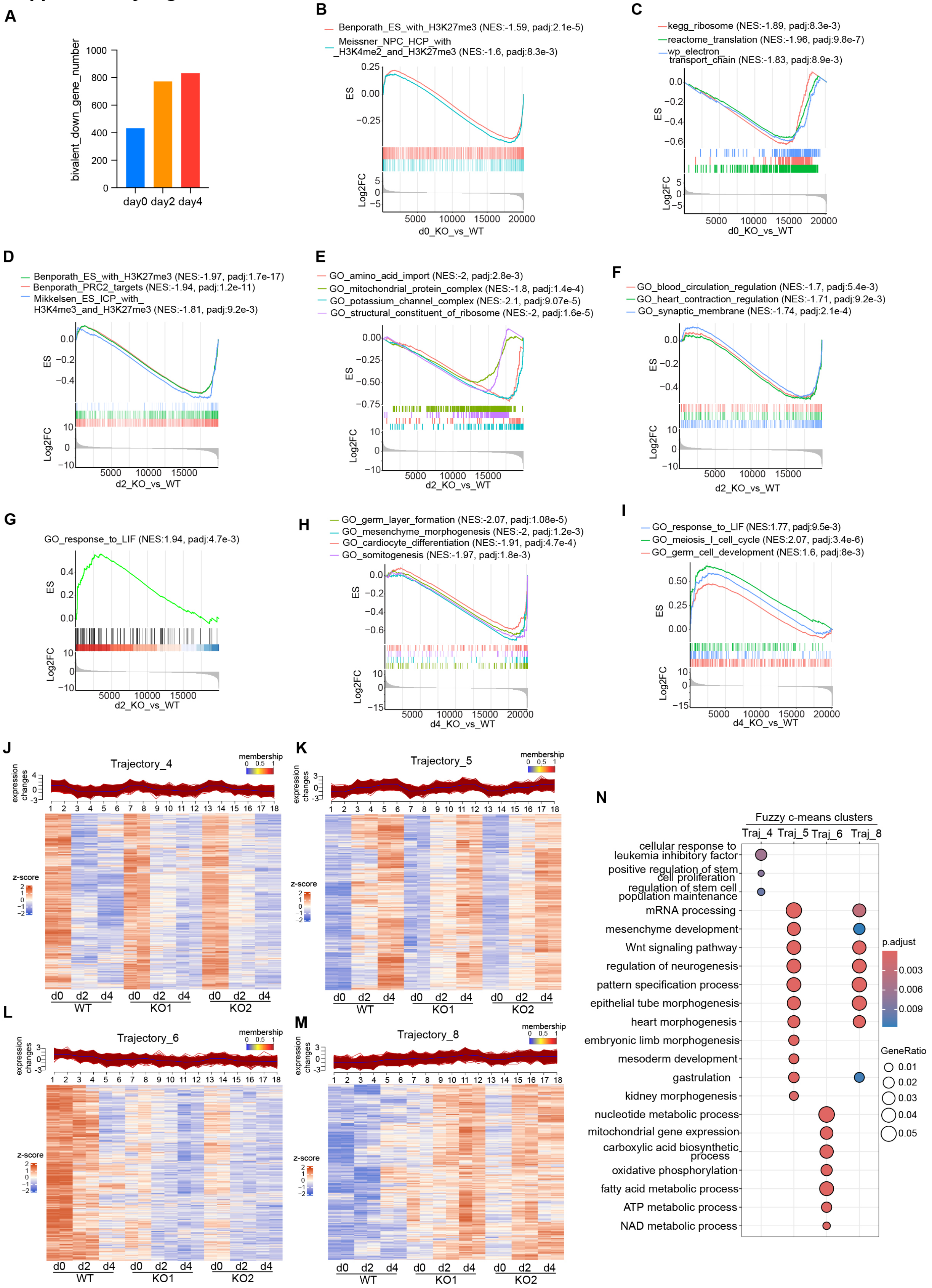

Supplementary Figure 3, Related to Figure 3.
A, Bar plot comparing the number of the significantly downregulated bivalent genes over time during mESC differentiation, based on RNA-seq.
B-I, GSEA showing enrichment of the indicated gene sets against a ranked RNA-seq gene list (**B-C**: KO vs. WT at day 0; **D-G**: KO vs. WT at day 2, **H-I**: KO vs. WT at day 4). NES, normalized enrichment score. *P* values were determined by an empirical phenotype-based permutation test and BH-adjusted for gene set size and multiple hypotheses testing.
J-M, Fuzzy c-means clustering of RNA-seq datasets identifies 8 clusters (named as Trajectory_1 to Trajectory_8) with distinct trajectory patterns. Traj_4 (**J**), Traj_5 (**K**), Traj_6 (**L**) and Traj_8 (**M**) are shown as line plots (top) and heatmaps (bottom) of z-score expression. Black lines in the line plots are cluster centroid; genes are colored by the degree of cluster membership; genes with max_membership > 0.9 are plotted.
N, Dot plot comparing GO terms enriched among the genes of Traj_4 (n=1538), Traj_5 (n=1587), Traj_6 (n=1244), Traj_8(n=1226). Dots are differentially colored and sized based on their corresponding BH-adjusted p values and gene ratio values, respectively. P values were calculated by hypergeometric test and BH-adjusted for multiple comparisons.

Supplementary Figure 4

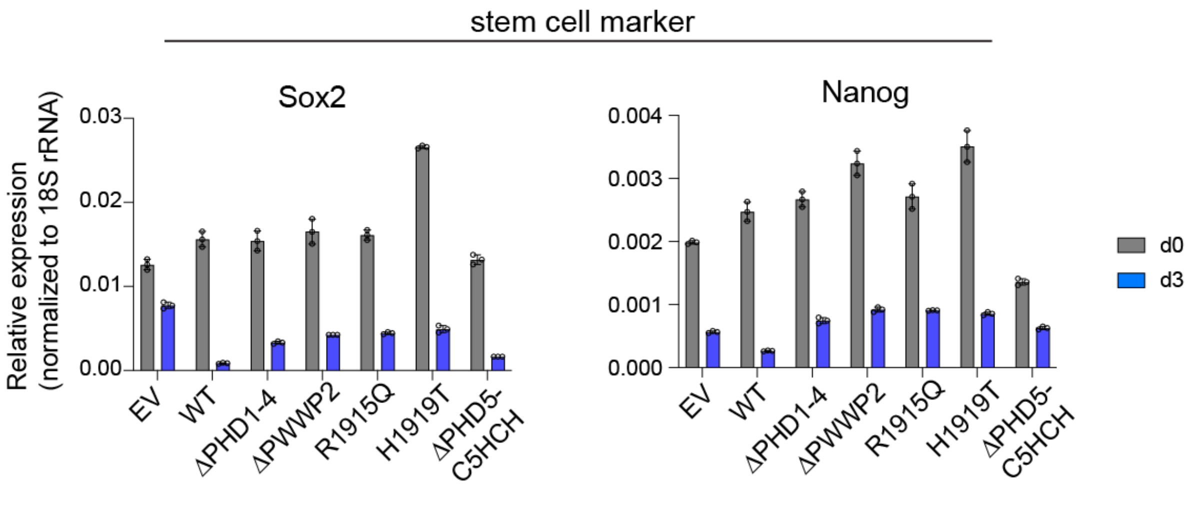

Supplementary Figure 4, Related to Figure 4.
RT-qPCR analysis of stem cell marker genes, Sox2 and Nanog, before or after a three-day differentiation of Nsd1-KO mESCs with the stably transduced Nsd1, WT or the indicated mutant. RT-qPCR signals from three independent experiments were normalized to those of 18S rRNA and presented as mean ± SD.

Supplementary Figure 5

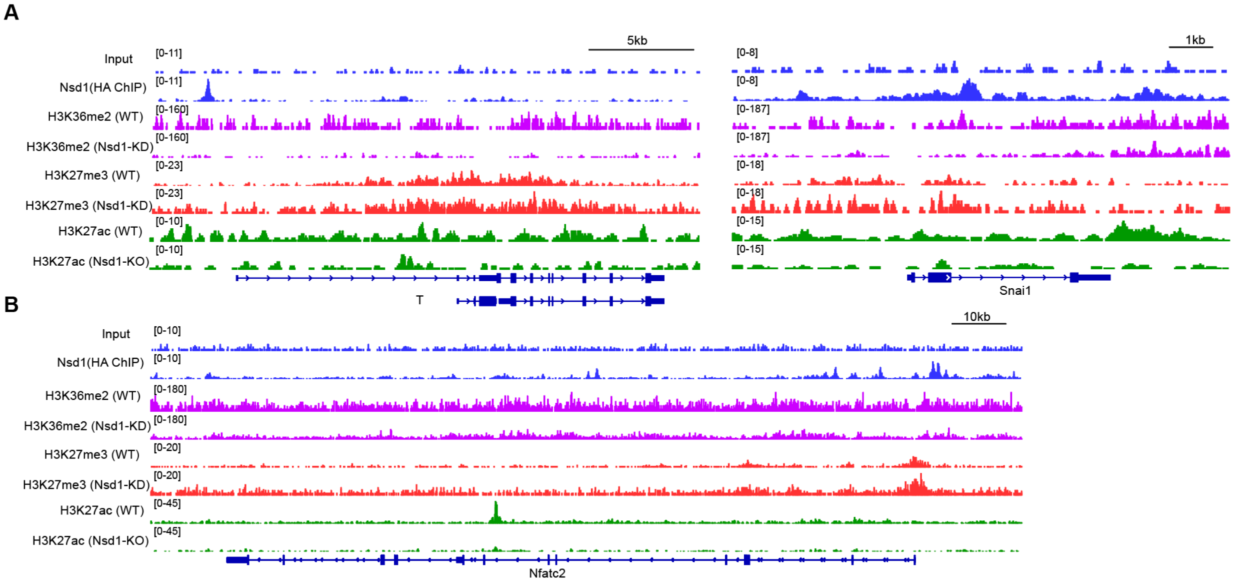

Supplementary Figure 5, Related to Figure 5.
A-B, IGV views of the indicated RPGC-normalized ChIP-seq signals at the cell lineage specification genes in either WT or Nsd1-KD/KO mESCs.
